# Marshall Hall, M. D.

**Published:** 1853-04

**Authors:** 


*Marshall Hall, M. D.* — It is stated that this distinguished physician is about to visit this country.

How rare is it that we have an opportunity of seeing the distinguished members of the medical profession of other countries on our own soil! Cannot some plan be devised by which they may be attracted to our shores, and, thus, a fair reciprocity, in this respect, be established?

Since the foregoing was penned, we find the following in the Boston Med. and Surg. Journal:

*Dr. Marshall Hall.*—Of course every medical reader is familiar with the name of this distinguished English author. He is now at Washington, accompanied by his lady and son. After visiting the South, he proposes to return and travel over the West extensively, and next season visit the Eastern States. Although considerably advanced in years, Dr. Hall is a laborious student, a close observer, and may justly be called one of the most celebrated medical writers of the age. We bespeak for him the attentions of the professional brotherhood wherever his steps may be directed.

